# Thyroid storm in pregnancy: a review

**DOI:** 10.1186/s13044-024-00190-y

**Published:** 2024-01-17

**Authors:** Vidhu Vadini, Prabhav Vasistha, Almog Shalit, Spyridoula Maraka

**Affiliations:** 1 Department of Internal Medicine, University of Arkansas for Medical Sciences, 4301 W. Markham St, Little Rock, AR 72205 USA; 2 Endocrine Unit and Diabetes Center, Department of Clinical Therapeutics, School of Medicine, Alexandra Hospital, National and Kapodistrian University of Athens, Athens, Greece; 3 Division of Endocrinology and Metabolism, University of Arkansas for Medical Sciences, 4301 W. Markham St, Little Rock, AR 72205 USA; 4 Section of Endocrinology, Central Arkansas Veterans Healthcare System, 4300 W. 7Th St, Little Rock, AR 72205 USA

**Keywords:** Thyroid storm, Pregnancy, Hyperthyroidism

## Abstract

**Background:**

Thyroid storm is a state of circulating thyroid hormone excess leading to multiorgan dysfunction and systemic decompensation. It typically occurs in the setting of poorly controlled hyperthyroidism and a precipitating illness or event. Management of thyroid storm in pregnancy poses unique diagnostic and therapeutic challenges.

**Main body:**

Thyroid storm is a clinical diagnosis characterized by hyperpyrexia, tachyarrhythmias, congestive heart failure, gastrointestinal and neuropsychiatric disturbances. However, diagnostic scoring systems have not been validated in pregnancy.

Treatment involves specialist consultation, supportive care, and pharmacological options such as anti-thyroid medications, beta blockers, iodine solutions, glucocorticoids, and cholestyramine. These must be adapted and modified in pregnancy to prevent fetal and maternal complications.

**Conclusion:**

There is a critical need to recognize thyroid storm during pregnancy and initiate proper medical interventions promptly.

## Introduction

Thyroid storm is a rare endocrine emergency. Due to the lack of universal diagnostic criteria, it is difficult to ascertain the true incidence of thyroid storm. Numerous studies have reported that between 5-16% of all patients admitted with thyrotoxicosis have thyroid storm [1–3]. Thyroid storm in pregnancy confers high maternal-fetal mortality and morbidity, if not treated promptly [4]. The incidence of overt hyperthyroidism in pregnancy ranges between 0.2%-0.9%, highest in the first trimester [4–8]. A population-based retrospective cohort study of a Canadian database reported the prevalence of thyroid storm as 2.97 per 1,000,000 pregnancies [9]. In-hospital mortality of thyroid storm has been reported between 3-11% [1–3]. This review discusses the clinical features, complications, and considerations in management of thyroid storm in pregnant patients.

## Causes and risk factors

The most common causes of hyperthyroidism in pregnancy are Graves' disease (0.1-1% of pregnancies) and human chorionic gonadotropin (hCG)-mediated hyperthyroidism due to gestational transient thyrotoxicosis (1-3% of pregnancies) [10, 11]. hCG shares an alpha subunit with the thyroid stimulating hormone (TSH) and there is 38% sequence identity in their beta subunits [12]. As a result, hCG stimulates the TSH receptor via molecular mimicry [13]. Other causes of hyperthyroidism in pregnancy include multinodular toxic goiter, toxic thyroid adenoma(s) [14], struma ovarii [15], factitious thyroid hormone intake, subacute thyroiditis, TSH secreting pituitary adenoma [16], hydatidiform mole [17–20], and functional thyroid cancer metastases [20]. Most cases of thyroid storm are due to an underlying diagnosis of hyperthyroidism secondary to Graves’ disease but can also occur in cases of toxic adenomas, multinodular goiters, or an hCG-secreting hydatidiform mole [21]. Untreated and uncontrolled hyperthyroidism is a major risk factor for the development of thyroid storm during pregnancy [4]. Similarly to the general population, thyroid storm in pregnancy can be precipitated by acute stressors such as infection, surgery, trauma, burns, hypoglycemia/diabetic ketoacidosis, and drugs/toxins (amiodarone, chemotherapy, aspirin toxicity, organophosphates or iodinated contrast) [22–26], but it can also be precipitated by pregnancy-associated conditions (anemia, pre-eclampsia, placenta previa, induction of labor/C-section, chorioamnionitis etc.) [4, 27–30].

## Clinical features

In general, the clinical features of thyroid storm in pregnancy do not differ from those in non-pregnant patients.

### Signs and symptoms

Patients with thyroid storm exhibit severe, life-threatening symptoms of hyperthyroidism. These include hyperpyrexia (> 103°F), tachycardia (usually > 140 bpm), cardiac arrhythmias, signs of congestive heart failure (shortness of breath, edema), nausea, vomiting, diarrhea, and neuropsychiatric disturbances such as confusion, restlessness, altered mentation, tremors, and seizures [3, 4, 30, 31].

### Laboratory/imaging findings

Measurement of serum TSH, free or total thyroxine (T4), and free or total triiodothyronine (T3) is warranted in patients who present with characteristic signs and symptoms of thyroid storm. Reference ranges of these hormones in pregnancy are assay and trimester dependent and differ from those in the general population [14]. The degree of elevation in free T4 does not correlate with the severity of thyroid storm, although this has not been shown specifically in the pregnant population. Elevated leukocyte count, hepatic transaminases, erythrocyte sedimentation rate/C-reactive protein, B-type natriuretic peptide, and creatine kinase may be seen [32–36].

When the etiology of hyperthyroidism is uncertain, TSH receptor antibodies (TRAbs), using third-generation assays like thyrotropin-binding inhibitory immunoglobulin (TBII) or thyroid-stimulating immunoglobulin (TSI), should be measured. TRAbs are positive in 96-97% of patients with Graves' disease, confirming its diagnosis [37]. Thyroid radionuclide imaging to differentiate Graves’ disease from thyroiditis is contraindicated in pregnancy [38]. Thyroid ultrasound with Doppler flow may be useful in experienced clinicians to distinguish Graves' disease (high blood flow) from painless thyroiditis (low blood flow) [39]. However, its utility in diagnosing hCG-mediated hyperthyroidism is unknown.

### Diagnosis

Thyroid storm is a clinical diagnosis. Diagnostic systems such as the Burch-Wartofsky Point Scale (BWPS) (Table 1) or the Japan Thyroid Association (JTA) criteria (Table 2) may be used to establish the diagnosis and evaluate the severity of the thyroid storm [30, 31], but have not been specifically validated in pregnancy. These systems differ in the number and type of parameters evaluated as well as their scoring methods. The BWPS is known for its higher sensitivity but lower specificity, while the JTA criteria may exhibit reduced sensitivity in diagnosis [40].

**Table 1:** Burch-Wartofsky Point Scale

**Thermoregulatory Dysfunction**	
Temperature (°F/°C)	
99 to 99.9 | 37.2 to 37.7	5
100 to 100.9 | 37.8 to 38.2	10
101 to 101.9 | 38.3 to 38.8	15
102 to 102.9 | 38.9 to 39.4	20
103 to 103.9 | 39.4 to 39.9	25
≥ 104.0 |≥ 40.0	30
**Central Nervous System Effects**	
Agitation	10
Delirium/Psychosis/Extreme lethargy	20
Seizure/Coma	30
**Gastrointestinal/hepatic Dysfunction**	
Diarrhea/nausea/vomiting/abdominal pain	10
Unexplained jaundice	20
**Cardiovascular Dysfunction**	
Heart rate	
99 to 109	5
110 to 119	10
120 to 129	15
130 to 139	20
≥ 140	25
Atrial fibrillation	10
Heart failure	
with pedal edema	5
with bibasilar rales	10
with pulmonary edema	15
**History of Precipitating Factors**	
Present	10
Absent	0

A score of > 45 or more is suggestive of thyroid storm, a score of 25–44 suggests impending thyroid storm and thyroid storm is unlikely if the score is < 25

**Table 2:** The diagnostic criteria for thyroid storm of the Japan Thyroid Association

**Prerequisite for diagnosis**		
Presence of thyrotoxicosis with elevated levels of free triiodothyronine (FT3) or free thyroxine (FT4)		
**Symptoms**		
1. Central nervous system (CNS) manifestations: restlessness, delirium, mental aberration/psychosis, somnolence/lethargy, coma (≥ 1 on the Japan Coma Scale or ≤ 14 on the Glasgow Coma Scale)		
2. Fever: ≥ 38 °C		
3. Tachycardia: ≥ 130 beats per minute or heart rate ≥ 130 in atrial fibrillation		
4. Congestive heart failure (CHF): pulmonary edema, moist rales over more than half of the lung field, cardiogenic shock, or New York Heart Association class IV or Killip ≥ Class III		
5. Gastrointestinal (GI)/hepatic manifestations: nausea, vomiting, diarrhea, or a total bilirubin level ≥ 3.0 mg/dL		
**Diagnosis**		
*Grade of thyroid storm*	*Combinations of features*	*Requirements for diagnosis*
TS1	First combination	Thyrotoxicosis and at least one CNS manifestation and fever, tachycardia, CHF, or GI/hepatic manifestations
TS1	Alternate combination	Thyrotoxicosis and at least three combinations of fever, tachycardia, CHF, or GI/hepatic manifestations
TS2	First combination	Thyrotoxicosis and a combination of two of the following: fever, tachycardia, CHF, or GI/hepatic manifestations
TS2	Alternate combination	Patients who met the diagnosis of TS1 except that serum FT3 or FT4 level are not available
**Exclusions and provisions**		
Cases are excluded if other underlying diseases are clearly causing any of the following symptoms: fever (e.g., pneumonia and malignant hyperthermia), impaired consciousness (e.g., psychiatric disorders and cerebrovascular disease), heart failure (e.g., acute myocardial infarction), and liver disorders (e.g., viral hepatitis and acute liver failure). Therefore, it is difficult to determine whether the symptom is caused by thyroid storm or is simply a manifestation of an underlying disease; the symptom should be regarded as being due to a thyroid storm that is caused by these precipitating factors. Clinical judgment in this matter is required		

*TS1* Definite thyroid storm, *TS2* Suspected thyroid storm

## Complications

Pregnancy is associated with a physiological increase in cardiac output and decrease in systematic vascular resistance and blood pressure. Pregnant patients with thyroid storm are at a higher risk of development of cardiomyopathy and heart failure. This can manifest in the form of pulmonary edema and pleural effusion [4, 29]. Tachyarrhythmias such as atrial fibrillation (Afib) and supraventricular tachycardia (SVT) can lead to diastolic dysfunction [41]. Life-threatening vascular complications such as arterial aneurysmal rupture can occur [42].

Acute kidney injury and/or abnormal liver function tests may be seen due to right heart dysfunction and hypovolemia. Electrolyte disturbances are common, because of possible rhabdomyolysis and gastrointestinal (GI) losses through vomiting and diarrhea.

Progression of thyroid storm can lead to neurological dysfunction, including agitation, encephalopathy, psychosis, stroke, seizure, and coma [30, 43, 44].

Obstetric complications such as pregnancy-induced hypertension, pre-eclampsia and eclampsia, preterm birth, low birth weight, placental abruption, fetal hyperthyroidism, and stillbirth can jeopardize maternal-fetal health [4].

## Management

### General measures

The management of thyroid storm (Table 3) should occur in the intensive care unit (ICU) with continuous cardiac and fetal monitoring involving endocrinologists and maternal-fetal medicine specialists [4, 45].

**Table 3:** Management of thyroid storm in pregnant patients

	Management of thyroid storm in pregnant patients
General measures	• Admit to ICU
	• Continuous cardiac and fetal monitoring^a^
	• Emergent enteral and intravenous access
	• Fluid resuscitation with crystalloids
	• Correction of electrolyte abnormalities (e.g., hypokalemia)
	• External cooling and antipyretic therapy to target euthermia (acetaminophen preferred over aspirin)
	• Search for underlying etiology
	• Avoid delivery^a^
Urgent	• Antithyroid medications
	1st line – PTU 600–1000 mg loading dose followed by 200–250 mg q4h
	2nd line – MMI 20 mg q6h
	• Steroids
	Hydrocortisone (300 mg IV loading dose, then 100 mg IV q8h) (preferred over dexamethasone^a^)
	• Beta blockers
	1st line—Esmolol loading dose of 250–500 μg/kg IV over 30 s, followed by an infusion at 50–100 μg/kg/minute 2nd line – Propranolol enteral (60–80 mg q4-6 h) or IV (1 mg over 10 min)
	Avoid atenolol^a^
After 1 h	SSKI (5 drops or 250 mg q6-8 h) or Lugol’s iodine (10 drops or 0.5 mL q8h) (lithium carbonate contraindicatedª)
Refractory thyroid storm	• Cholestyramine 4 g q6h
	• Plasmapheresis –place patient in left lateral position to avoid IVC compression^a^
	• Consider emergent thyroidectomy
After resolution	Continue PTU (if in the 1st trimester) or switch to MMI (if in the 2nd trimester)ª
	Continue to monitor for antithyroid medication toxicity

*PTU* Propylthiouracil, *MMI* Methimazole, *SSKI* Saturated Solution of Potassium Iodide; *q* every, *IVC* Inferior vena cava, *IV* Intravenous, *h* hours

^a^Steps marked indicate the steps which are different from conventional management of thyroid storm in non-pregnant patients

Initial supportive measures include securing intravenous and enteral access, oxygen therapy to maintain SpO2 greater than 90% and endotracheal intubation for airway protection (if needed). If sedation is required, barbiturates may be used because they increase the catabolism of thyroid hormones, but they are considered category D by the United States (US) Food and Drug Administration (FDA) [46, 47]. There are few reports comparing benzodiazepines with other anxiolytic agents during pregnancy. Midazolam is theoretically superior to lorazepam due to the teratogenic effects observed in animal studies of lorazepam. Propofol crosses the placenta and may be associated with neonatal respiratory depression. Data on the clinical use of propofol for pregnant critically ill patients are limited to case reports, so its use should be limited until more prospective data are available [48, 49]. Crystalloids should be administered for fluid resuscitation to replace GI losses through vomiting/diarrhea and insensible losses through diaphoresis and fever. Electrolyte disturbances such as hypokalemia should be corrected. Thiamine may be administered empirically [43, 44]. Temperature management is done to target euthermia with external cooling and antipyretic therapy. Acetaminophen is preferred over aspirin because aspirin can theoretically displace thyroid hormone from the serum binding site and increase the levels of free thyroid hormone [14, 45].

Close monitoring is required to prevent hypoglycemia due to depletion of hepatic glycogen stores. Delivery should be avoided as per the American College of Obstetrics and Gynecology guideline [50]. Patients will need to be maintained on prenatal vitamins without iodine.

Expeditious search for the underlying cause should be attempted and antibiotics should be administered early if an infection such as chorioamnionitis is suspected [51].

Complications such as congestive heart failure require co-management with a cardiologist with cautious use of diuretics. Tachyarrhythmias such as AFib/SVT should be aggressively managed. Consideration should be given to inotropes or mechanical support devices for patients with severe heart failure refractory to medical treatment [52].

### Pharmacological treatment options

#### Beta blockers

Immediate treatment with a beta blocker controls the signs/symptoms associated with hyperadrenergic state such as tachyarrhythmias, hypertension, and hyperpyrexia. Beta blockers should be used cautiously in patients with reactive airway disease or peripheral vascular disease. Pregnant patients are at a higher risk for development of congestive heart failure; hence beta blockers should be used cautiously.

Esmolol is a beta-1 blocker administered intravenously. Esmolol is recommended by the JTA due to its short half-life of 9 min since pregnant patients are particularly vulnerable to developing cardiogenic shock with administration of beta blockers. Esmolol can be given as a loading dose of 250–500 μg/kg IV, followed by an infusion at 50–100 μg/kg/minute [14, 31].

Propranolol is a non-selective beta blocker which can be given enterally (60–80 mg every 4-6 h) or intravenously (1 mg over 10 min IV) if cardiac dysfunction is considered highly unlikely. It is recommended by the American Thyroid Association because at high doses it also inhibits peripheral conversion of T4 to T3 through inhibition of type 1 deiodinase [22, 37]. However, it has a duration of action of 6-12 h and has been associated with circulatory collapse [53, 54]. It is relatively contraindicated in reactive airway diseases such as asthma.

Atenolol can cause fetal growth restriction and should be avoided [55, 56].

#### Thioamides

Immediate treatment with thioamides such as propylthiouracil (PTU) and methimazole (MMI) is started to inhibit de novo thyroid hormone synthesis.

PTU is the drug of choice for the initial management of thyroid storm in pregnancy irrespective of trimester due to its inhibition of type 1 deiodinase (decreasing peripheral conversion of T4 to T3). PTU is given enterally as a loading dose of 600-1000 mg followed by 200-250 mg every 4 h. A study comparing PTU vs MMI found similar reductions in serum T4 but markedly reduced T3 in the PTU group (45% vs 10-12% within 24 h) [57]. MMI may be given orally at a dose of 20 mg every 6 h if PTU is contraindicated. Notably, findings from a recent large observational study support interchangeable use of these medications [58]. Intravenous and rectal administration of thioamides has also been described [59–62].

After resolution of thyroid storm, it is recommended to continue on PTU if the patient is in the 1st trimester, because MMI use has been associated with an increased incidence of severe congenital abnormalities such as craniofacial malformations (e.g., scalp epidermal aplasia [aplasia cutis], facial dysmorphism, and choanal atresia) [63]. PTU is also associated with birth defects, but they tend to be less severe and may not be diagnosed immediately after birth [64]. However, after resolution of thyroid storm, treatment with MMI is preferred in the 2nd and 3rd trimester since the important period of organogenesis is completed and there have been reports of PTU-associated fulminant hepatotoxicity [65]. Side effects common to both PTU and MMI include dermatological (pruritus, alopecia, skin pigmentation, urticaria), GI (nausea, vomiting), arthralgias, myalgias, and a lupus-like reaction (including vasculitis) [45]. Serious side effects such as agranulocytosis and liver failure can occur. The frequency of agranulocytosis is similar in patients receiving MMI (0.35%) and PTU (0.37%). However, their relative frequency in pregnant patients has not been elucidated [66]. In a cohort study conducted in Taiwan, hepatitis was more common in MMI/carbimazole users (3.17/1000 person-years) than PTU users (1.19/1000 person-years); however, the risk of acute liver failure and cholestasis was similar [57, 67]. Similar data in the US population or pregnant patients are lacking.

#### Iodine solutions

Iodine solutions induce the Wolff-Chaikoff effect, a transient inhibition of iodine organification and thyroid hormone release. These must be administered at least an hour after thioamide to avoid the Jod-Basedow effect (increased T4 formation and release) [30].

Various preparations used include Saturated Solution of Potassium Iodide (SSKI) (5 drops or 250 mg every 6-8 h) and Lugol’s iodine (10 drops or 0.5 mL every 8 h). Lugol’s iodine can also be added to intravenous fluids for patients unable to take oral medications and without enteral access. Lithium carbonate is contraindicated in pregnancy [4, 68].

Side effects are uncommon, but local esophageal or gastric irritation can occur.

#### Glucocorticoids

Pregnancy is associated with an altered set point of the hypothalamus-pituitary-adrenal axis and tissue resistance to the effects of cortisol. Thyroid storm, despite being a state of physiological stress, is associated with inappropriately normal or low serum cortisol. Administration of glucocorticoids helps to reverse this relative adrenal insufficiency. In addition, glucocorticoids decrease the peripheral conversion of T4 to T3 and may directly impact the underlying autoimmune process if the thyroid storm is due to Graves’ disease.

Hydrocortisone (300 mg IV loading dose, then 100 mg IV every 8 h) may be preferred over dexamethasone (2-4 mg IV every 8 h) as dexamethasone can cross the placenta [14, 37]. If dexamethasone is chosen, formulations free of alcohol, benzyl alcohol, or sodium sulfite should be used.

#### Cholestyramine

Cholestyramine is a bile acid sequestrant that reduces the enterohepatic circulation of T4 and T3 and enhances their elimination. It is given orally in the dose of 4 g every 6 h [69]. Cholestyramine interferes with the absorption of fat-soluble vitamins even in the presence of vitamin supplementation and therefore, regular prenatal supplementation may not be adequate. Thus, it is classified as FDA pregnancy category C. Despite lack of established safety data, benefit should outweigh risks in thyroid storm [70].

### Plasmapheresis

Physical removal of thyroid hormone can be attempted through plasmapheresis or charcoal plasmaperfusion [71–74] for refractory cases or if there are contraindications to thionamides. It works through extracorporeal removal of plasma proteins with bound T3/T4 [45].

Potential safety issues in pregnancy include fetal distress from transient hypovolemia and hypotension, hypofibrinogenemia, and hypogammaglobulinemia. Nadler’s equation needs to be modified to adjust for the plasma expansion. Pregnant patients should lay upon the left lateral decubitus position to avoid compression of the inferior vena cava (IVC) [75].

The effects of plasmapheresis are transient, lasting 48-72 h, and hence its use is usually reserved for rapid pre-operative preparation prior to thyroid surgery or in patients who are intolerant or refractory to thioamides. Due to the effect of plasmapheresis on coagulation, delivery should be avoided for at least 24 h following the procedure, if possible [75].

### Emergency thyroidectomy

Thyroidectomy may be performed as a last resort measure if medical management fails [71].

## Conclusion

Thyroid storm during pregnancy is a rare entity but can be destructive to both the mother and the fetus. Thus, prompt diagnosis and treatment are vital. The clinical manifestations and management plan are largely similar to non-pregnant cases, however appropriate considerations are necessary. In view of the physiologic changes of pregnancy, the diagnostic reference values are adjusted according to the trimester and certain complications are more commonly described. Additionally, caution should be employed during treatment selection to protect the developing fetus. Opportunities for studies with low risk of bias on the subject are scarce, considering the frequency and nature of the disorder, however, efforts to construct evidence-based guidelines are necessary to improve patient care and prevent maternal-fetal complications and mortality.

## Data Availability

Data sharing is not applicable to this article as no datasets were generated or analyzed during the current study.

## Abbreviations

Afib: Atrial fibrillation
BWPS: Burch-Wartofsky Point Scale
CHF: Congestive heart failure
CNS: Central nervous system
FDA: Food and Drug Administration
FT3: Free triiodothyronine
FT4: Free thyroxine
GI: Gastrointestinal
HCG: Human chorionic gonadotropin
ICU: Intensive care unit
IV: Intravenous
IVC: Inferior vena cava
JTA: Japan Thyroid Association
MMI: Methimazole
PTU: Propylthiouracil
SSKI: Saturated Solution of Potassium Iodide
SVT: Supraventricular tachycardia
T3: Triiodothyronine
T4: Thyroxine
TBII: Thyrotropin-binding inhibitory immunoglobulin
TRAbs: Thyrotropin receptor antibodies
TSH: Thyroid stimulating hormone
TSI: Thyroid-stimulating immunoglobulin
TS1: Definite thyroid storm
TS2: Suspected thyroid storm
US: United States
